# Fish Welfare in Recirculating Aquaculture Systems (RAS): The Imperative for Environmental Enrichment (EE)

**DOI:** 10.3390/ani16040635

**Published:** 2026-02-17

**Authors:** Lorenzo Fruscella, Annamaria Passantino, Benz Kotzen

**Affiliations:** 1 School of Design, University of Greenwich, Park Row, London SE10 9LS, UKb.kotzen@gre.ac.uk (B.K.); 2 Department of Veterinary Sciences, University of Messina, Via Palatucci Annunziata, 98168 Messina, Italy

**Keywords:** fish welfare, recirculating aquaculture systems, environmental enrichment, allostasis, behavior

## Abstract

Aquaculture has become the fastest-growing food production sector worldwide, recently surpassing wild-capture fisheries in total output. This rapid expansion underscores the need to ensure sustainability through robust animal welfare standards. Recirculating Aquaculture Systems (RAS) are increasingly adopted due to their advantages in biosecurity, water efficiency, and production control. However, these systems often expose fish to highly artificial and environmentally impoverished conditions, which may negatively affect their welfare. This article examines fish welfare in RAS through the lens of environmental enrichment (EE), arguing that its implementation is essential to address behavioral, cognitive, and physiological needs. By integrating EE into RAS design and management, it is possible to move beyond traditional homeostatic welfare models focused solely on stress reduction toward an allostatic framework that emphasizes adaptability, agency, and positive experiences. Such an approach supports the concept of providing farmed fish with a “life worth living.” The paper highlights the ethical and practical implications of enrichment strategies and emphasizes their potential role in promoting sustainable and welfare-oriented aquaculture practices.

## Simple Summary

As aquaculture represents the fastest-growing food production sector globally, having recently surpassed wild-capture fisheries in output, ensuring sustainable practices through adequate welfare standards is paramount. Although Recirculating Aquaculture Systems (RAS) are increasingly adopted due to their operational advantages, these systems frequently expose teleosts to highly artificial and sensory-deprived environments, potentially compromising biological fitness and welfare. This paper argues that implementing environmental enrichment (EE) could significantly enhance fish well-being by transitioning from traditional homeostatic welfare models to those based on allostasis, where stability is achieved through change, thereby providing animals with a “life worth living”.

## 1. Introduction

Aquaculture is the fastest-growing food sector worldwide, with over 50% of all fish consumed coming from farms [1]. Globally, per capita consumption of fish rose from 9 kg to 20.5 kg between 1961 and 2018, with 3.3 billion people acquiring 20% of their animal protein from fish [2]. Despite this expansion, the industry faces critical welfare challenges, including disease outbreaks, poor water quality, overcrowding, improper handling, and the inability of fish and other aquatic animals to express natural behavioral repertoires [3,4].

Farmed fish comprise very diverse taxonomic groups compared with terrestrial animals [5], with fewer than 20 terrestrial species being farmed worldwide [6], whilst over 500 aquatic species are farmed in aquaculture, 350 of which are finfish [7]. The range of fish species farmed for human consumption is reflected in the variety of aquaculture systems used, including raceways, ponds, cages, and recirculating aquaculture systems (RAS). The locations where such systems are implemented include the open sea, along coasts, in the intertidal zones, in lakes, and in ponds. Land-based RAS offer a variety of advantages, most notably an exceptionally high level of control of water parameters, which has resulted in their widespread adoption. Recent improvements in RAS technology, especially in oxygenation and water treatment, facilitated greater stocking densities and consequently productivity. However, this intensification has also prompted public awareness and induced policymakers in Europe to pay more attention to fish welfare, prompted by both ethical considerations and the prospect of improving the quality and standards of aquaculture and fisheries technologies [8]. One way of recognizing and enforcing welfare standards originates in organic aquaculture, which is characterized by an all-inclusive approach to food production that conserves natural resources, adopts best environmental practices, and maintains biodiversity [9]. As noted by Ahmed et al. [10] (p. 1), organic aquaculture is a system that was developed *‘as a potential substitute to address environmental constraints faced by intensive aquaculture’*. Organic standards in the European Union (EU) are considered to be among the strictest in the world, with the new Regulation (EU) 848/2018 on organic agriculture and aquaculture [11], which became effective in January 2021, putting an even greater focus on fish welfare. In this regard, standards for organic fish production in the EU could be the catalyst needed for the introduction of major measures for the improvement of welfare in fish.

The main tenets of organic certification of aquatic animals in the EU include the forbidden use of RAS, the restricted and sustainable use of energy, and the use of measures that make the farming environment as similar as possible to the natural environment of the cultured animal species [12]. RAS, with their innovative low water exchange rates and high environmental controls, are gaining popularity over more traditional industrial systems [13]. However, the structurally minimalist and aseptic nature of RAS provides the cultured fish with what may be considered to be an impoverished habitat that is often very different from their natural environment, and for this reason, RAS are excluded from being able to achieve organic certification in the EU. The European Market Observatory for Fisheries and Aquaculture Products (EUMOFA) notes that ‘*fish welfare is not necessarily ensured in RAS, and several projects have experienced mass mortality, due to design errors or technical difficulties of the water recirculation’* [14] (p. 1). Nonetheless, RAS offers significant advantages compared with traditional aquaculture methods such as ponds, including complete control of water parameters (temperature, aeration, pH, salinity, current, and light), and minimal chances of escapees into the external environment [15]. Moreover, if integrated with aquaponics, RAS can offer decreased or even zero waste pollution, taking into account the animal waste that is processed and converted into nutrients for the plants [16].

As Gerber et al. [17] point out, environmental enrichment-the practice of increasing the complexity of the holding environment of animals-could compensate for some of the negative features of RAS, namely the highly sterile and barren environment and the noise from pumps, aerators, and filters. However, as pointed out by Arechavala-Lopez [18], the neglect of environmental enrichment in aquaculture is largely rooted in the ongoing and still inconclusive discourse regarding fish sentience and their capacity for nociception and subjective suffering.

The word ‘fish’ encapsulates a wide and highly diverse group of vertebrates; in fact, a fish is broadly defined as ‘an animal that lives in water, is covered with scales, and breathes by taking water in through its mouth’ [19]. Fish encompass over 60% of all known vertebrate species and are traditionally divided into three extant classes: Agnatha (hagfish and lampreys), Chondrichthyes (sharks and rays), and Osteichthyes (bony fish, the most numerous fish in terms of species) [20]. According to FishBase [21], more than 26,000 of the over 33,000 total fish species are teleosts, a subdivision of bony fish, which have been around for over 500 million years [22]. Teleosts are found in almost every type of aquatic environment and exhibit a large range of physiological and behavioral traits; the great majority of the over 500 species of fish cultured worldwide are teleosts [23]. Therefore, it is important to approach generalization across different fish species with some caution. Fish are unique amongst other vertebrate farmed animals, since their deaths are recorded by weight rather than by the individual number of animals killed. In fact, fish killed for human consumption are both so numerous and so poorly documented that every account is highly approximate at best. Best estimates report that every year between 40 and 140 billion fish are slaughtered in commercial fish farms, and between 830 and 2.4 trillion fish are wild-caught globally [20]. Although knowledge and implementation of fish welfare is lagging compared with terrestrial vertebrates, public as well as governmental awareness and concerns about fish welfare are increasing [17], with growing evidence that consumers express concern for the welfare conditions of farmed fish and may be willing to pay price premiums for products derived from higher-welfare systems [24].

This study offers a review of current fish welfare issues, including the question of fish sentience and the current welfare status of finfish in EU legislation, and makes suggestions on the types of modifications that could be implemented in RAS in order to improve fish welfare. Although RAS are used for culturing a variety of aquatic animal taxa, including crustaceans (prawns, lobsters, crabs) and molluscs (bivalves, octopuses), this review will focus on finfish, and particularly on bony fish (Superclass: Osteichthyes), given their predominance and widespread use in RAS.

## 2. The Importance of Fish Welfare

It has been argued that, given how different fish are from terrestrial animals, it is impossible to transfer the knowledge acquired regarding the latter, which has progressed significantly in the past several decades, to fish; in fact, the challenges presented by fish are not comparable [25]. Possibly the most prominent difference in the study of welfare between terrestrial and aquatic animals is the lack of recognized and clearly observable behavioral patterns. In fact, the aqueous environment of fish poses significant challenges in terms of the direct observation of possible welfare indicators [17].

Historically, the fish have been three different non-mutually exclusive concepts under which animal welfare has been defined [26]:

(1) Feeling-based definitions: These regard farmed animals as sentient beings able to experience feelings and consequently suffer emotionally. Good feeling-based welfare is considered to require a reduction in negative experiences, such as fear or stress, and the substantial presence of positive ones;
(2) Function-based definitions: These consider animal welfare to be a direct function of good health and normal biological growth and functioning, with a focus on the ability of animals to adapt to their current environments. This concept has been criticized for being reductionist, with Ashley [27] (p. 2) claiming physical health to be ‘*the most universally accepted measure of welfare and* […] *undoubtedly required for good welfare*’, adding that ‘*for many, good welfare goes beyond just physical health and also involves a lack of mental suffering*’;
(3) Nature-based definitions: These are based on the inherent biological nature of animals and their expression. In nature-based welfare practices, good welfare is fulfilled if the animals are able to lead natural lives and can engage in natural behaviors.

## 3. Fish Sentience and Capacity for Pain

Human interactions with fish are generally limited compared with those involving terrestrial animals. Most people rarely observe fish in their natural habitats or witness the full range of their species-specific behaviors. This limited familiarity has likely shaped societal attitudes towards fish and influenced perceptions of their cognitive and emotional capacities. Indeed, attitudes toward different animal taxa are often closely linked to perceived intelligence and behavioral complexity [28].

Historically, fish have been portrayed within a linear evolutionary framework that positioned vertebrates along a gradient from “primitive” to “advanced,” culminating in humans. Within this paradigm, fish were frequently characterized as evolutionarily basal vertebrates with simple brains and correspondingly limited behavioral and neural capacities [29]. However, this hierarchical model is increasingly regarded as an oversimplification of vertebrate evolution and neurobiology.

The extent and nature of sentience in fish remain subjects of active scientific debate. In ethical discourse, sentience is commonly defined as the capacity to experience subjective states such as pleasure and pain [30]. Consciousness is a broader construct, encompassing sentience alongside cognitive processes such as awareness of internal and external stimuli, aspects of self-representation, and varying degrees of self-awareness [31]. Although intelligence and sentience are conceptually distinct, animals exhibiting advanced cognitive abilities are often considered more likely to possess sentient capacities [32,33]. Recent research on the cognitive abilities of fish suggests that they are, in fact, much more intelligent than once thought. Fish possess a similar use of geometric cues as birds and rats, exhibit complex social skills, and can quickly learn to avoid aversive stimuli [29]. They can learn to differentiate between individuals, as in the case of gobies, and several clades like the cichlids and cyprinids provide parental care [29], and, according to some recent studies, fish may even be self-aware [34,35]. Overall, there is strong evidence that finfish experience emotions and possess long- and short-term memory. In other words, they are sentient [31,36]. Whilst it is logical to assume fish experience pain, the topic remains controversial. Addressing it would require significant changes to fish husbandry in order to satisfy higher welfare standards.

### The Debate on Pain Perception

The sentience of mammals and birds, and consequently their capacity to experience pleasure and pain, is widely recognized in European scientific and legislative contexts. In contrast, the ability of fish to experience suffering has historically been questioned or dismissed, despite increasing evidence that fish share key physiology and neurochemistry features with other vertebrates [20]. According to the European Food Safety Authority (EFSA)’s Panel on Animal Health and Welfare (AHAW) [22] (p. 10), ‘vertebrates, fish, birds and mammals share a similar general brain structure’. The fish brain, like that of mammals, comprises a forebrain (including diencephalon and telencephalon), midbrain, and hindbrain. However, it differs in overall organization and lacks the extensive cerebral cortex that characterizes the mammalian forebrain [37,38]. While the mammalian cerebral cortex, particularly the six-layered neocortex, is often associated with higher cognitive processing, the absence of a structurally homologous region in fish has been central to arguments denying their capacity for conscious pain experience. Nonetheless, AHAW [22] (p. 3) concludes by stating that ‘there is scientific evidence to support the assumption that some fish species have brain structures potentially capable of experiencing pain and fear’. The stress responses of fish are similar to those of mammals: under stress, fish release noradrenalin and adrenalin, which result in several physiological effects, including increased ventilation and heart rate, and in response to emotional stimuli, they produce cortisol [39]. Serotonin, dopamine, oxytocin, and other transmitter substances associated with emotional reactions in humans have also been found in fish [40,41], as have neural pathways, nociceptors, and brain areas for the processing of pain [42].

The case for the exclusion of fish from other basic welfare rights seems to be based on the long-held premise that fish are non-sentient animals, and consequently incapable of feeling pain, as laid out in the 2014 paper by Rose et al. ‘Can Fish Really Feel Pain?’, where it is concluded that ‘the behavioral and neurobiological evidence reviewed shows fish responses to nociceptive stimuli are limited and fishes are unlikely to experience pain’ [43] (p. 97). Rose et al. [43] report the presence of numerous insufficiencies in the methods used for the identification of pain, particularly when trying to distinguish subconscious pain (nociception) from the conscious feeling of pain. They also note the lack of a neocortex in the fish brain, which in mammals is thought to play an significant role in the neural mechanism that processes sensory perception and emotion, and which produces the subjective experience of suffering. Furthermore, Rose et al. [43] claim that an extensive literature exists which shows normal activity, including feeding immediately or shortly after surgery. This is confirmed by the absence of C-fiber nociceptors in most teleost fish, and a total absence in the elasmobranchs studied to date, which are the most predominant and widespread type of nociceptors in mammals and are the ones responsible for producing agonizing pain in humans [43]. Overall, the authors claim that there is a lack of acceptable supporting evidence and neurological feasibility for the presence of consciousness in fish, as well as their ability to perceive pain.

As argued by Eurogroup for Animals [20], the arguments put forward by Rose et al. [43] are Cartesian in that they are oversimplified, as they ignore the fact that fish physically react to damage and injury, which is all reduced to an unconscious response. Moreover, birds lack a neocortex yet are broadly recognized as sentient and legally protected in many jurisdictions. Huntingford et al. [33] suggest that complex behavioral repertoires and cognitive capacities in fish make it plausible that they are capable of experiencing some form of suffering, even if its qualitative nature differs from that in humans.

Research by Sneddon et al. [44] concluded that fish nociceptors are remarkably similar to those in humans, and that, in fact, the application of analgesics in fish has been observed to reduce the symptoms of pain. Painkillers such as morphine have been found to work on fish; moreover, the fish brain produces painkillers known as endogenous opioids—this happens in other vertebrates too and can be taken as further proof that fish do indeed feel pain [37]. Sneddon et al. [44] observe that fish react consciously to pain, have the required anatomy to feel pain, and suffer as a result of pain being inflicted, thus debunking the claim that a neocortex is necessary to experience pain and providing evidence that fish have the capacity to suffer. Research by Sneddon et al. [44] in particular has encouraged a discussion in the public and political domains on the importance of the protection of fish from suffering. Brown [29] claims that fish could not survive as the behaviorally and cognitively complex animals they are if they did not feel pain, suggesting that the physical detection of pain should not be separated from its cognitive or emotional response, since they comprise the same combined system that has evolved to minimize the chances of harm, protect the individual, and maximize the chances of survival [42]. In fact, consciousness is an evolutionary adaptation that provides some animals with significant life advantages, where feeling pain and responding appropriately to it are of utmost importance to survival and could have even preceded the evolution of complex cognitive processing [45].

One of the more remarkable forms of evidence that fish may experience pain at a cognitive level is that exposure to noxious stimuli has been shown to induce cognitive impairments, such as attention deficits, suggesting a conscious processing of pain [29]. Broom, Sneddon et al., and Brown [46,47,48] all state that considering nociception as detached from emotional response is old-fashioned and counterproductive, as nociception and pain belong to the same integrated system. Considering the fundamental role of this system is in harm avoidance, it is likely to have originated in early vertebrate lineages and to be highly conserved across taxa [49,50]. Brown [29] and Barton & Venditti [51] challenge the claim by Rose et al. [43] that the human neocortex constitutes the exclusive center of consciousness and that, because fish lack a homologous structure, they cannot be conscious. They argue that the human neocortex has not undergone uniquely dramatic evolutionary changes and that there is no compelling evidence to consider it more intrinsically linked to consciousness than other brain regions. In support of this view, Tononi & Edelman [52] propose the ‘dynamic core hypothesis’ of consciousness, according to which no single anatomical locus can be identified as its seat; rather, conscious experience emerges from patterns of functional neuronal connectivity, which are not constrained by strict biofilanatomical proximity.

A further advocate of the idea that fish are incapable of feeling pain is Key [53]. Basing his thesis on the bioengineering principle that ‘structure determines function’, the author concludes that, since fish lack the necessary neuro-cytoarchitecture, structural connectivity, and microcircuitry for the neural processing necessary for feeling pain, they are anatomically incapable of feeling pain. Braithwaite & Droege [54] disagree with this thesis, arguing that it is based on the false assumption that the processing of human pain is the reference point for assessing pain in other animals and that convergent evolution makes it possible for different structures to perform the same function. This means that fish could have evolved different anatomical and physiological mechanisms that allow them to feel pain. Braithwaite & Droege [54] add that Key [53] does not recognize the presence of ongoing debates on the neuroanatomy of consciousness in humans. In fact, it is likely that attentional amplification and global integration are essential elements of consciousness. Thus, Braithwaite & Droege [54] argue that the issue is not whether fish possess an insula or a cortex, but whether they possess the functional capability for attentional amplification and global integration.

Anatomically, whilst the evolutionary and developmental trajectory of the brain of fish differs from that of other vertebrates, it possesses numerous analogous structures that execute very similar functions. Overall, based on this evidence, the amount of cognitive complexity that is displayed by fish seems to be comparable to that of most other vertebrates, and fish are indeed sentient and able to feel distress and pain in a manner similar to humans and other mammals [29].

## 4. Fish Welfare Challenges in RAS

RAS are land-based facilities designed for intensive fish production at both hatchery and growing stages. They minimize water exchange using integrated mechanical filtration and biofiltration units designed to mitigate toxicity through nitrification. Although RAS are often described as closed systems, periodic water replenishment is required to compensate for evaporation and cleaning losses. Water is continuously treated, purified, and recycled to maintain conditions suitable for fish health.

Originally derived from a combination of wastewater treatment and aquaculture technology [55], RAS have been commercially implemented for over four decades, with rapid technological advancements occurring particularly in the past twenty years. Water treatment in RAS typically involves multiple sequential processes to ensure depuration and reuse while maintaining appropriate physicochemical parameters. Core components generally include [55]:

- Mechanical devices to filter out solid particles from the water, which include uneaten feed, bacterial flocs, and fish feces;
- Biofilters, which oxidize ammonia excreted by the fish into less toxic nitrate via nitrifying bacteria;
- Gas exchange devices, which remove dissolved CO_2_ produced by the fish and add the oxygen needed by the fish and the nitrifying bacteria.

Additional technologies, such as UV irradiation, water disinfection, protein skimming, ozonation, and denitrification systems, are increasingly adopted to improve microbial control and manage fine particulates and nitrate accumulation [55].

RAS offers several advantages compared with traditional flow-through ponds or cage systems. These include adjustable stocking densities, enhanced control of water quality, improved environmental biosecurity, reduced exposure to predators, precise pharmaceutical administration, minimal escape risk [15], and the possibility of waste valorization (e.g., integration into aquaponic systems). Furthermore, RAS may reduce environmental discharge and enable closer monitoring of fish health and behavior, as parameters such as pH, dissolved oxygen, temperature, and photoperiod can be continuously measured and adjusted [55,56].

Despite such advantages, RAS environments are typically structurally barren and differ markedly from the ecological conditions experienced by fish in the wild. System design has historically prioritized economic efficiency and operational practicality, often with limited consideration of behavioral and cognitive needs [18]. This ecological simplification partly explains why certain RAS operations may not meet organic certification criteria [20,57]. As indicated in a 2009 briefing paper by animal welfare organization Compassion in World Farming, practices commonly applied in intensive aquaculture operations (including RAS), such as stripping of broodstock, handling of live fish, vaccinations, crowding, size grading, starvation, loading, and transportation, all expose cultured fish to a wide range of stressors that their wild counterparts are not subjected to [58]. To date, welfare assessment in RAS has largely been approached from a production-oriented perspective, with the primary objective of maximizing growth and minimizing mortality. Consequently, welfare evaluation has traditionally relied on indicators such as growth performance, stress biomarkers, disease prevalence, stocking density (biomass per unit volume), and water quality parameters [55,59].

Because fish condition in RAS is highly dependent on system performance, the main goal of welfare in RAS has been to achieve the best systems and parameters that result in minimal disease, stress, and mortality, and the highest productivity. Water quality studies in this regard have mostly focused on [55]:

- The concentration limits of nitrogenous compounds;
- The concentration limits of dissolved CO_2_;
- The effects of ozonation;
- The accumulation of recalcitrant compounds.

While acute stress responses represent adaptive mechanisms evolved to cope with environmental threats and do not necessarily indicate poor welfare, chronic or repeated stress can result in detrimental tertiary effects that compromise health and productivity [27,60]. Accordingly, welfare protocols have largely targeted the prevention of prolonged stress exposure. Regulatory and advisory bodies emphasize the importance of environmental conditions in safeguarding welfare. The UK government’s Farm and Animal Welfare Committee (FAWC), later renamed Animal Welfare Committee (AWC) in 2019, states that fish ‘*need sufficient space to show most normal behavior with minimal pain, stress and fear*’ [55], reiterating the perceived importance of stocking density, together with water quality, in the majority of fish welfare protocols. Whilst stocking density is widely regarded as a key benchmark for assessing welfare in aquaculture, cultured species exhibit marked interspecific variations in stocking density thresholds and behavioral responses, reflecting substantial differences in tolerance, social dynamics, and environmental preferences. For example, rainbow trout (*Oncorhynchus mykiss*) exhibit decreased growth rates when cultured at higher stocking densities [61], whilst sea bass (*Dicentrarchus labrax*) and juvenile gilthead sea bream (*Sparus aurata*) show higher stress levels at increased densities [62]. On the other hand, Arctic char (*Salvelinus alpinus*) grow better and feed more when stocked at high densities [63]. This has been addressed by some private fish welfare standards, such as G.A.P.’s “Animal Welfare Standards for Farmed Atlantic Salmon v1.0”, which imposes maximum stocking densities, as well as environmental enrichment practices, for salmon in RAS [64].

Beyond stress-related indicators, fish welfare is also critically affected by skeletal deformities, which constitute a major welfare concern, as demonstrated in gilthead seabream (*Sparus aurata*), where higher stocking densities during the pre-ongrowing phase were associated with a significantly increased prevalence of cranial and vertebral anomalies [65].

Although water quality and stocking density are major drivers of welfare in RAS, other environmental stressors intrinsic to intensive recirculating facilities also play a significant role. In particular, persistent anthropogenic noise and vibrations originating from pumping and aeration units, blowers, and filtration units, as well as lighting regimes with different intensities, spectra, and photoperiods, can also influence fish behavior, stress physiology, circadian regulation, growth, and health [66,67]. Furthermore, disease dynamics are a key welfare and management concern in recirculating aquaculture systems (RAS), as viral disease outbreaks can cause rapid and severe mortality events. High stocking densities, chronic stress, and inadequate husbandry or biosecurity can increase host susceptibility and facilitate pathogen transmission, while the closed and highly interconnected nature of RAS can amplify outbreak impacts; explicitly incorporating viral disease risk into RAS welfare assessments therefore strengthens both their biological relevance and practical value [68].

## 5. Environmental Enrichment (EE): A New Paradigm

### 5.1. Beyond the Five Freedoms: The Allostasis Model

Traditionally, fish welfare has been framed according to the “Five Freedoms” proposed by the World Organization for Animal Health (WOAH) [69]. This paradigm focuses on mitigating negative states, such as hunger, fear, and disease, to ensure physical health and safety. Although internationally recognized and integrated into legislation (e.g., the UK Animal Welfare Act 2006), the model is increasingly viewed as obsolete. Critics argue that the mere absence of suffering does not equate to positive welfare and that the “Freedoms” rely on a rigid homeostatic view that oversimplifies the relationship between stress and well-being [70,71].

In response to these conceptual limitations, the “Five Domains” model was developed to incorporate the promotion of positive affective states alongside the mitigation of negative ones. By systematically evaluating nutrition, environment, health, and behavior, this framework integrates their combined impact on the animal’s subjective “Mental State.” The core rationale is that good welfare requires providing opportunities for rewarding, goal-directed behaviors that generate pleasure or interest [72].

A further conceptual advancement is represented by the shift from homeostasis—defined as the maintenance of internal constancy—to allostasis, which emphasizes achieving stability through adaptive change. Within the allostatic model, welfare is compromised not only by excessive or chronic stress (hyperstimulation) but also by insufficient environmental input (hypostimulation), such as that encountered in barren or monotonous systems. Optimal welfare is theorized to occur within an intermediate range of stimulation, often described as “eustress,” where manageable environmental challenges activate motivational and reward systems and promote effective coping strategies [73,74].

Building on these principles, the concepts of animal “agency” and “positive effective engagement” have gained prominence in welfare science. These perspectives conceptualize animals as active agents capable of interacting with, modifying, and deriving meaningful experiences from their environment, rather than as passive recipients of husbandry. Providing solvable environmental challenges can foster behavioral expression, cognitive flexibility, and a sense of control, thereby supporting higher tiers of Quality of Life (QoL), ranging from a “life worth living” to a “good life” [75,76].

**Figure 1:**
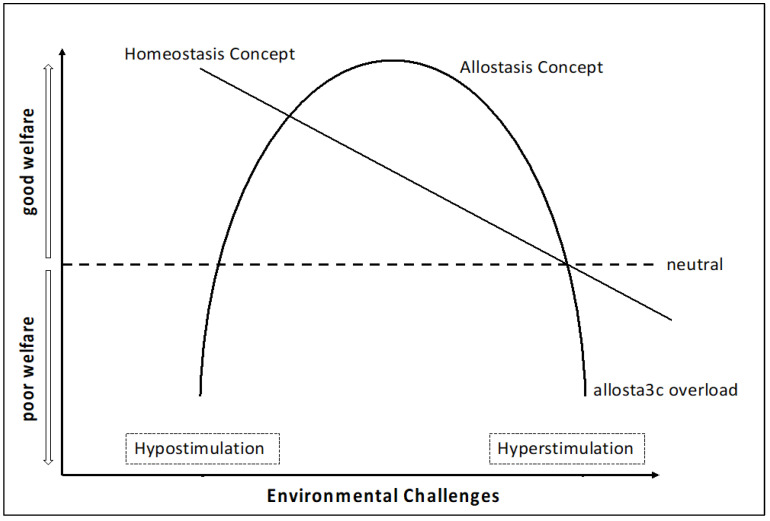
Animal welfare level relating to environmental challenges as shown by the outdated ‘stability through constancy’ linear homeostasis concept versus the new ‘constancy through change’ hyperbolic allostasis concept, according to which too few or too many environmental challenges are suboptimal and result in hypostimulation and hyperstimulation, respectively; an intermediate level of environmental challenges is necessary for good welfare. Adapted from Raposo de Magalhães et al. [71] and Korte et al. [78].

Within this theoretical framework, environmental enrichment is identified as the primary tool to achieve these high-welfare standards in aquaculture. By increasing environmental complexity and mimicking natural stimuli, enrichment provides the “eustress” necessary for fish to exercise cognitive flexibility and behavioral adaptability (Figure 1). Recent studies, such as Spiliopoulos et al. [77], demonstrate that occupational enrichment, such as exercise regimes and training in stressor predictability, enhances stress resilience. In this context, stress is not inherently negative but becomes a manageable and rewarding challenge that strengthens coping capacity, reinforces agency, and ultimately contributes to an improved QoL.

### 5.2. Defining and Implementing Environmental Enrichment (EE)

Despite growing public concern regarding aquatic welfare, the complexity of the holding environment remains a frequently neglected dimension in intensive aquaculture. Current academic discourse identifies the mapping of species-specific needs and the design of targeted interventions as the most critical research frontier for modern fish welfare [4].

Environmental Enrichment (EE) is defined as a deliberate increase in environmental complexity aimed at mitigating maladaptive traits and enhancing psychological and physiological well-being [79,80]. According to Young [81], the primary objectives of EE include increasing behavioral diversity, reducing abnormal behaviors, and enhancing the animal’s capacity to cope with environmental challenges.

Importantly, EE simultaneously addresses multiple welfare paradigms, encompassing feeling-based (affective states), function-based (biological functioning), and nature-based (expression of species-typical behaviors) approaches. These strategies are particularly applicable to RAS, where tank parameters can be precisely modified. The most common forms of EE are categorized as follows:

- Physical enrichment, which includes the addition of structures such as substrates (e.g., gravel, sand), shelters, and tank covers to increase habitat complexity;
- Sensory enrichment, which concerns the brain and sensory organs, including auditory, visual, tactile, taste, or olfactory stimuli;
- Dietary/nutritional enrichment, including variations in feed type and delivery frequency to encourage natural foraging strategies and feeding behaviors [82];
- Social enrichment, which involves adding/removing direct or indirect interactions and contacts with conspecifics as well as with humans to stabilize social hierarchies;
- Occupational enrichment, which involves increasing environmental variation in order to alleviate psychological and physical monotony. This can encompass tools that provide animals with challenges or that encourage exercise [82]. Whilst previously considered secondary for fish [17], recent evidence suggests that occupational tools, such as controlled water currents, significantly enhance growth, flesh quality, and stress resilience [18].

A meta-analysis of 147 studies covering 82 species concluded that enriched environments significantly improve welfare outcomes compared to barren tanks [83]. Specifically, in the physical domain, the provision of structural complexity and shelters has been shown to influence stress physiology and behavioral expression in Atlantic salmon, including reduced cortisol responses and increased shelter use under hatchery conditions [84]. Sensory enrichment studies further demonstrate that environmental color and visual background can affect reproductive physiology and behavior in Nile tilapia [85]. Dietary and occupational enrichment approaches have been shown to stimulate natural foraging behavior and environmental engagement in cultured fish species [18]. Finally, social environment modulation, including group structuring and density-related interactions, has been linked to differences in coping styles, aggression, and stress responsiveness in farmed fish populations [86]. Reflecting this growing body of evidence, certification schemes such as Friend of the Sea^®^ have begun incorporating EE-related criteria into their welfare standard [18].

Nevertheless, a substantial gap remains between experimental findings and large-scale industrial implementation. To date, EE in commercial aquaculture has often been adopted primarily to boost productivity, such as utilizing substrates for flatfish or artificial seaweed for spawning. To achieve organic certification in RAS, a shift toward more holistic EE protocols is deemed essential [58].

It is argued that the aquaculture sector should look toward the advancements made by modern zoos and aquariums [70], where EE is already an established pillar of positive welfare management [87,88,89,90]. Further species-specific research is required to refine enrichment practices and ensure that the diverse biological requirements of farmed fish are adequately met within commercial production systems.

### 5.3. Challenges in Formulating Species-Specific Environmental Enrichment Guidelines

A significant barrier to the standardization of EE is the immense phylogenetic diversity of cultured finfish, which spans various orders, families, and genera. This biological variety necessitates highly differentiated EE approaches tailored to the specific ecological niches of each species. Furthermore, a substantial knowledge gap persists between laboratory-based observations and farm-level applications; many studies yield contradictory results, likely due to inadequate experimental designs regarding life stages or specific rearing conditions [17,18].

To manage this complexity, it has been proposed to categorize fish into functional groups, following the FAO model [91]:

- Group 1 (Cyprinids): Carps, barbels, and other cyprinids;
- Group 2 (Cichlids): Tilapias and other cichlids;
- Group 3 (Siluriformes): Catfish;
- Group 4 (Salmonids): Salmons, trouts, and smelts

While pragmatic, such broad groupings may not adequately capture species-specific variability. Even closely related taxa can exhibit divergent behavioral and physiological responses to identical enrichment stimuli. For instance, increased environmental complexity has been reported to reduce aggression in *Tilapia rendalli* but to exacerbate it in *Oreochromis niloticus* [92,93]. These findings suggest that enrichment guidelines should be informed by phylogenetically coherent clades—such as those recognized under the International Code of Zoological Nomenclature (e.g., Family or Order)—while remaining sensitive to species-level ecological and behavioral distinctions.

Several initiatives are currently attempting to bridge knowledge gaps and harmonize welfare assessment. The European Cooperation in Science and Technology (COST) Action on Welfare of Fish in European Aquaculture [94] focuses on developing scientifically proven welfare indicators for industrial use. Similarly, the Fair-Fish Database provides an open-access platform translating ethological and physiological data into practical welfare criteria for more than 500 farmed species [95].

Despite these efforts, there is a clear need for a holistic, standardized assessment system analogous to the Welfare Quality^®^ (WQ) program used in terrestrial livestock. A fish-specific WQ model could evaluate welfare across four key principles: feeding, housing, health, and behavior. Within such a framework, EE protocols would be integrated under the “housing” principle to ensure an unbiased, evidence-based evaluation of the rearing environment [96]. It is also important to note that EE should not be viewed solely within the “housing” domain, as its effects often extend to behavioral expression and, where relevant, feeding strategies. Framing EE across these interconnected principles would more accurately reflect its functional role and align with the multidimensional structure of contemporary welfare assessment approaches.

To facilitate the transition of theoretical advances into practical application, future research should prioritize the following areas:

- Diversification of EE modalities: expanding investigation of undertested strategies, specifically dietary, social, and occupational enrichment;
- Broadening the species focus: shifting the research focus from laboratory models (e.g., *Danio rerio*) to high-output aquaculture species, such as grass carp (*Ctenopharyngodon idella*) and various pleuronectiforms (flatfish);
- Methodological standardization: establishing consistent trial durations and incorporating ontogenetic variables (e.g., age, sex, developmental stage), as short-term behavioral measures may not reliably predict long-term welfare outcomes [80];
- Commercial integration: assessing the feasibility and efficacy of EE within intensive commercial settings, with a specific focus on establishing welfare criteria tailored for RAS.

Although the applied evidence base remains relatively nascent, recent studies conducted under hatchery and commercial conditions demonstrate that certain enrichment interventions—such as substrate addition and structural complexity—can be operationally implemented with reported benefits for behavioral development, stress coping, and production-relevant outcomes [97,98]. Expanding such production-scale research will be essential for establishing system-specific welfare criteria for intensive aquaculture.

## 6. Conclusions

Contrary to historical misconceptions of fish as biologically simplistic, a substantial body of contemporary evidence demonstrates that they lead complex lives, possess emotional capacities, and exhibit sophisticated responses to pain and stress. Framed within the modern welfare paradigm of allostasis, this review argues that aquaculture facilities should provide adequate environmental stimuli to meet the evolutionary and ethological requirements of each species, thereby promoting adaptive regulation rather than mere survival.

While RAS offers an extraordinary level of control over water parameters compared to traditional aquaculture systems, they remain largely sterile environments designed primarily to optimize productivity. Integrating RAS with EE practices may represent a promising pathway towards innovative aquaculture systems that address not only the physiological prerequisites for physical health but also the sensory and cognitive needs underpinning a ‘life worth living’.

The transition to commercial-scale EE, however, presents substantial challenges. While certain enrichment strategies have been implemented to support reproduction, the complex trade-off between welfare enhancements and operational constraints—including intensified maintenance, elevated capital expenditure, and hygiene risks—remains insufficiently investigated [17]. Specifically, the accumulation of organic waste within enrichment structures may compromise water disinfection protocols, posing health risks if not mitigated through appropriate engineering solutions [18]. Furthermore, evidence suggests that EE can induce phenotypic changes; for instance, rainbow trout (*Oncorhynchus mykiss*) reared in structurally complex organic environments exhibit distinct morphological characteristics in head and trunk regions compared with conspecifics raised in intensive raceways [99]. It should also be observed that behavioral repertoires and ecological requirements shift markedly across ontogeny, implying that environmental enrichment strategies should be adapted to larval, juvenile, and adult stages [80].

Enhancing fish welfare also carries practical implications beyond ethical considerations. Recent evidence indicates that welfare-enhancing conditions such as reduced chronic stress, greater environmental predictability, and opportunities for species-specific behaviors can improve physiological robustness, immune competence, and stress coping ability, thereby reducing disease susceptibility and mortality. These improvements may translate into better growth performance, feed efficiency, and resilience to husbandry and environmental challenges, thereby contributing to production stability and the long-term sustainability of aquaculture systems [86].

In conclusion, the implementation of EE in aquaculture should be evidence-based, species-specific, and life-stage appropriate, using natural history and behavioral ecology as a benchmark while carefully accounting for the technical constraints of intensive systems [18]. Given the diversity of farmed aquatic species, future research should prioritize rigorous evaluation of the economic feasibility, husbandry compatibility, and long-term production outcomes of EE in industrial contexts, ensuring that welfare enhancements are both biologically effective and commercially sustainable.

## Data Availability

No new data were created or analyzed in this study. Data sharing is not applicable to this article.
